# Comparative analysis of mesenchymal stem cell and embryonic tendon progenitor cell response to embryonic tendon biochemical and mechanical factors

**DOI:** 10.1186/s13287-015-0043-z

**Published:** 2015-05-09

**Authors:** Jeffrey P Brown, Thomas V Galassi, Matteo Stoppato, Nathan R Schiele, Catherine K Kuo

**Affiliations:** Department of Biomedical Engineering Tufts University, Science and Technology Center, 4 Colby Street , Medford, MA 02155 USA; Cell, Molecular & Developmental Biology Program Sackler School of Graduate Biomedical Sciences, Tufts University School of Medicine, 145 Harrison Avenue, Boston, MA 02111 USA

## Abstract

**Introduction:**

Advances in tendon engineering with mesenchymal stem cells (MSCs) are hindered by a need for cues to direct tenogenesis, and markers to assess tenogenic state. We examined the effects of factors involved in embryonic tendon development on adult MSCs, and compared MSC responses to that of embryonic tendon progenitor cells (TPCs), a model system of tenogenically differentiating cells.

**Methods:**

Murine MSCs and TPCs subjected to cyclic tensile loading, transforming growth factor-β2 (TGFβ2), and fibroblast growth factor-4 (FGF4) *in vitro* were assessed for proliferation and mRNA levels of scleraxis, TGFβ2, tenomodulin, collagen type I and elastin.

**Results:**

Before treatment, scleraxis and elastin levels in MSCs were lower than in TPCs, while other tendon markers expressed at similar levels in MSCs as TPCs. TGFβ2 alone and combined with loading were tenogenic based on increased scleraxis levels in both MSCs and TPCs. Loading alone had minimal effect. FGF4 downregulated tendon marker levels in MSCs but not in TPCs. Select tendon markers were not consistently upregulated with scleraxis, demonstrating the importance of characterizing a profile of markers.

**Conclusions:**

Similar responses as TPCs to specific treatments suggest MSCs have tenogenic potential. Potentially shared mechanisms of cell function between MSCs and TPCs should be investigated in longer term studies.

## Introduction

Tendons transmit muscle-derived forces to bone to enable skeletal movement. Unfortunately, these tissues suffer ~15 million musculoskeletal injuries annually in the USA [1]. Due to the poor innate healing ability of tendons, surgical intervention is the primary approach to repairing injured tendon despite substantial failure rates, limited long-term function recovery, donor site morbidity with autologous transplants, and risk of infections [2,3]. These significant drawbacks have motivated efforts to engineer replacement tendon with mesenchymal stem cells (MSCs) [4-9].

Adult MSCs are attractive for tissue regeneration strategies as they have the potential to differentiate toward various musculoskeletal lineages, including osteogenic, chondrogenic and adipogenic, in response to established lineage-specific cues. However, such cues have not been identified for tenogenic differentiation, and tissue engineering approaches to tenogenically differentiate MSCs have not achieved functional tendons [4-14]. This may be in part because evaluation of tenogenic differentiation is challenged by limited knowledge of how tenogenically differentiating cells should behave. Scleraxis (Scx) is the only known tendon-specific marker that is expressed during early development and sustained throughout tissue formation [15]. However, Scx expression levels do not vary in embryonic tendon progenitor cells (TPCs) between developmental stages [16]. Furthermore, mice with a mutation in the Scx gene have defects in only a subset of tendons, indicating Scx is not a master regulator of tendon differentiation [17]. Recognizing these limitations, we recently examined how a profile of tendon markers, including Scx, late marker tenomodulin (Tnmd), and other relevant but non-specific markers (transforming growth factor (TGF)β2, collagen type I (Col I) and elastin (Eln)), respond to embryonic tendon cues [16].

We identified TGFβ2, and combinations with fibroblast growth factor-(FGF)4 and loading, as potential *in vitro* tenogenic cues based on upregulation of Scx and modulation of other tendon markers in embryonic TPCs, a model system of tenogenically differentiating cells [16]. Understanding how embryonic progenitor cells respond to developmental factors has been successful in establishing stem cell differentiation programs for other lineages. For example, protocols to direct chondrogenesis of adult MSCs are based on methods that utilize embryonic cartilage development factors to chondrogenically differentiate embryonic mesenchymal limb bud cells [18,19]. Factors to guide stem cell differentiation are selected based on their ability to induce marker expression patterns similar to that exhibited temporally by embryonic mesenchymal progenitor cells during development [20-25]. In contrast, how MSCs respond to treatments in comparison with embryonic cells that are committed to the tendon lineage (that is, TPCs) has not been investigated.

The need for mechanical loading for adult tendon homeostasis has motivated application of dynamic tensile loading as a primary cue to tenogenically differentiate MSCs. However, reports on the effectiveness of loading on tenogenesis have been inconsistent [6-8,10,26], and thus the efficacy of dynamic tensile loading to tenogenically differentiate MSCs is unclear. Developmentally, mechanical loading seems critical for tendon formation [27,28], as muscle paralysis during embryonic chick development resulted in malformed tendons [29-31]. However, paralysis may also have contributed to aberrant tendon formation by altering soluble factors secreted by muscle, such as FGF4 [32,33]. We reported mechanical loading alone had little effect on embryonic TPC behavior, but that specific loading and growth factor combinations differentially regulated tendon marker gene expression [16]. Interactions between growth factors and dynamic loading could play a key role in tenogenesis.

Tendon engineering strategies with MSCs have used growth factors involved in adult tendon wound healing [13,14], including TGFβ1, insulin-like growth factor, platelet-derived growth factor, epidermal growth factor, and FGF2 [34], despite their potential roles in the formation of scarred tendon with aberrant biochemical composition, organization and mechanical properties [35]. In contrast, embryonic tendon development involves different factors, including FGF4 and TGFβ2 [32,33,36-38]. Though we demonstrated FGF4 and TGFβ2 influence embryonic TPC activity [16], the ability for these factors to tenogenically differentiate adult MSCs has not been reported.

We hypothesized that MSCs would mimic TPCs in their response to tendon development factors. To test this hypothesis, we treated mouse adult MSCs and embryonic day (E) 14 TPCs with combinations of TGFβ2, FGF4 and mechanical loading, and assessed proliferation and gene expression. Our findings provide insight into MSC tenogenic potential and the utility of embryonic tendon factors to guide adult MSC differentiation toward a tenogenic lineage *in vitro*.

## Methods

All materials were from Invitrogen (Carlsbad, CA, USA) unless otherwise specified.

### Adult mouse bone marrow mesenchymal stem cell harvest

Four-month-old male Scx-green fluorescent protein (GFP) mice [39] were sacrificed by CO_2_ asphyxiation and decapitation with Tufts University Institutional Animal Care and Use Committee approval. The hind limbs were skinned, and femurs and tibias were dissected and washed in sterile phosphate-buffered saline (PBS) without MgCl_2_/CaCl_2_. Bone ends were removed and marrow was flushed with PBS. Cell suspensions were treated with red blood cell lysis buffer (Roche, Indianapolis, IN, USA), pelleted, washed with PBS, and resuspended in growth medium (GM) of Dulbecco’s modified Eagle medium with 10% fetal bovine serum (FBS) and 1% penicillin/streptomycin. Cells were plated at 1 × 10^6^ cells/cm^2^ and cultured at 37°C and 5% CO_2_. Three independent MSC pools, isolated by plastic adherence [7], were expanded to passage 3.

### Embryonic mouse tendon progenitor cell harvest

E14 embryos were harvested from pregnant Scx-GFP mice and staged [40] with Tufts University Institutional Animal Care and Use Committee approval. Limbs were isolated, minced, incubated under agitation at 200 rpm in 1% type II collagenase in PBS at 37°C for 45 minutes, and neutralized with GM. Cell suspensions were passed through a 40-μm cell strainer (BD Biosciences, San Jose, CA, USA), pelleted, washed in PBS, re-suspended in GM, plated at 1 × 10^4^ cells/cm^2^, and cultured at 37°C and 5% CO_2_. Three independent limb cell pools were harvested. Cells were trypsinized when 80% confluent and sorted on the basis of GFP signal using a MoFlo Legacy cell sorter (Beckman Coulter, Brea, CA, USA) at 488 nm excitation and collected by a 530/40 filter. TPCs were expanded to passage 1–2.

### Growth factor treatment and mechanical loading

TPCs and MSCs were seeded at 2 × 10^4^ cells/cm^2^ on Col I-coated Uniflex® plates (Flexcell International, Hillsborough, NC, USA) and incubated in GM for cell attachment. After 48 hours (day (D)0), GM was replaced with basal (control) medium (BM; Dulbecco’s modified Eagle medium, 1% FBS and 1% penicillin/streptomycin), or BM supplemented with 100 ng/mL rhFGF4 and/or 1 ng/mL rhTGFβ2 (PeproTech, Rocky Hill, NJ, USA), and cyclically loaded under uniaxial tension with 1% sinusoidal strain at 0.5 Hz for 1 hour/day, as previously described [16]. Static controls were treated identically, without cyclic loading. Medium was replaced after 48 hours.

### Cell proliferation

Cells were fixed for 20 minutes in 10% phosphate-buffered formalin, stained with 4′,6-diamidino-2-phenylindole nucleic acid dye, and imaged using an inverted Leica DM IL fluorescent microscope and DFC340 FX camera (Leica Microsystem, Buffalo Grove, IL, USA). Three fields per well (left, middle, and right) were imaged for each condition and cell pool. Nuclei were counted using Image J (National Institutes of Health, Bethesda, MD, USA).

### Quantitative polymerase chain reaction

MSCs and TPCs were harvested on D0 and D3 for RNA isolation. Cells were homogenized in TRIzol reagent and total RNA was isolated. Samples were reverse-transcribed using the Superscript III First Strand Synthesis kit. Quantitative (q)PCR was performed with Brilliant II SYBR Green qPCR master mix (Agilent, Wilmington, DE, USA) on a Stratagene Mx3000P multiplex qPCR system (Agilent). Previously optimized mouse-specific primers for Scx, Tnmd, Col I, Eln, TGFβ2 and 18 s were used [16]. Fold change was calculated as 2^–ΔΔCT^.

### Statistical analysis

Results were obtained from three independent cell pools and are shown as mean ± standard deviation. Statistical analyses were performed using GraphPad Prism (GraphPad Software Inc., San Diego, CA, USA). Treatment effects were evaluated using a two-way analysis of variance with Tukey's *post-hoc* test or Student’s *t*-test, and considered statistically significant when *P* < 0.05. qPCR data were log-transformed before statistical analysis and plotted as fold difference values (2^-ΔΔCT^).

## Results

### Effects of treatments on mesenchymal stem cells

**Figure 1:**
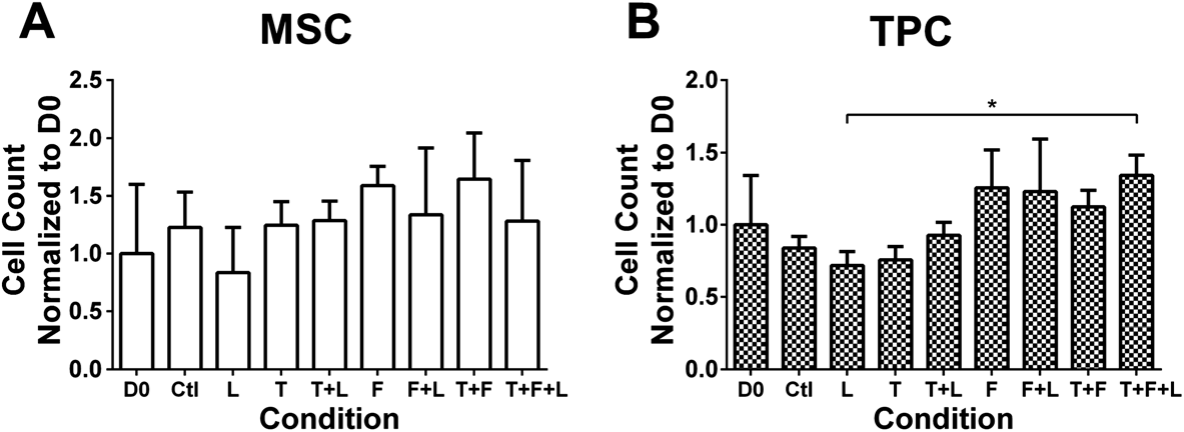
Mesenchymal stem cell (MSC) and tendon progenitor cell (TPC) proliferation as a function of growth factor treatments and loading. Effects on MSC and TPC proliferation on day (D)3 (normalized to D0) of treatment with combinations of mechanical loading (L), transforming growth factor (TGF)β2 (T), and fibroblast growth factor-(FGF)4 (F) treatment. Left column shows D0 data. **(A)** MSC proliferation was not significantly affected by any treatment. **(B)** TPC proliferation was not significantly affected by any treatment, but there was a significant difference between loading and TGFβ2 + FGF4 + loading groups on D3. *P < 0.05.

**Figure 2:**
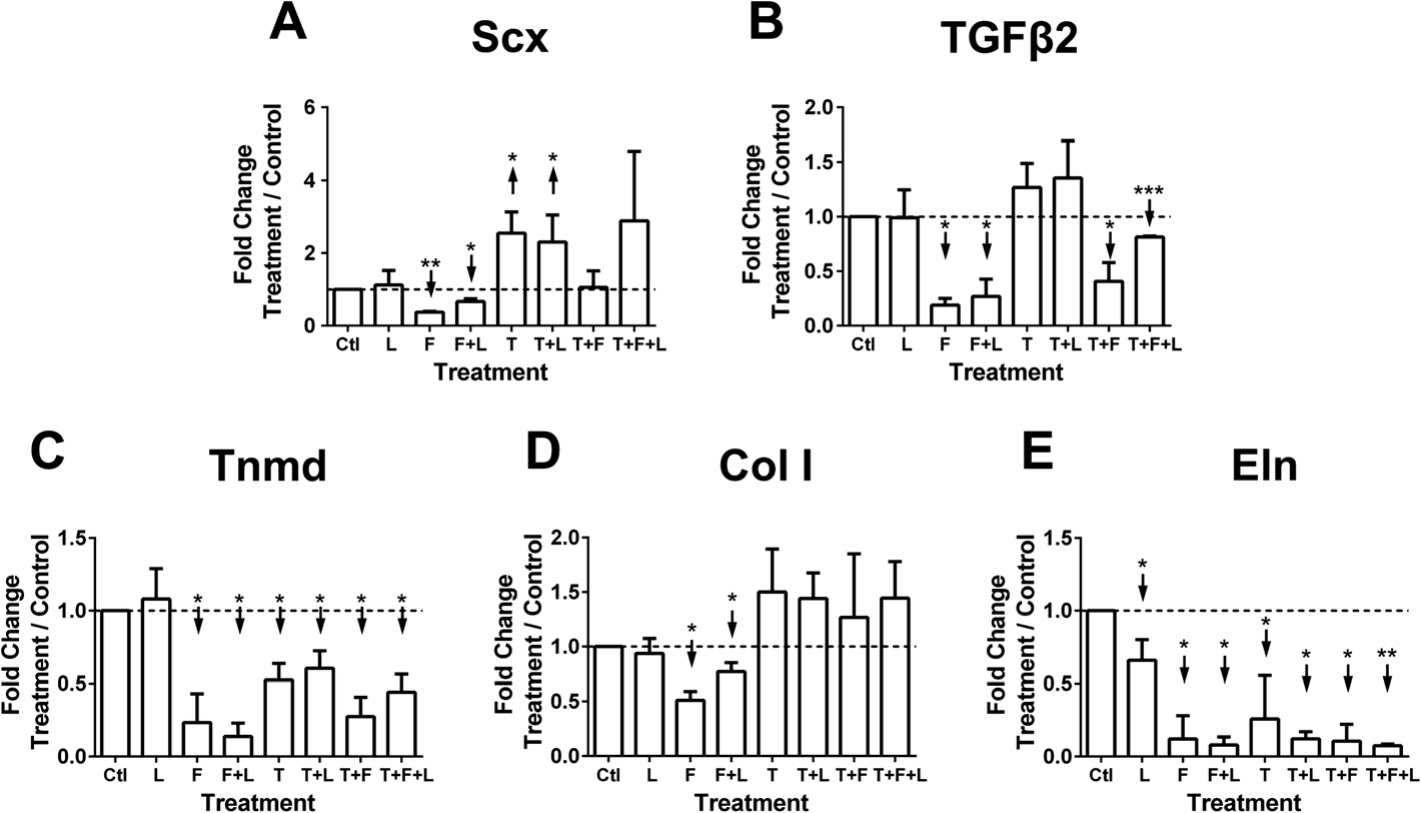
Mesenchymal stem cell (MSC) tendon marker gene expression as a function of growth factor treatments and loading. MSC gene expression on day (D)3 of treatment with combinations of mechanical loading (L), transforming growth factor (TGF)β2 (T), and fibroblast growth factor-(FGF)4 (F). Dashed horizontal line = 1 indicates control condition. **(A)** Scleraxis (Scx) was significantly downregulated by FGF4 and FGF4 + loading, and upregulated by TGFβ2 and TGFβ2 + loading. **(B)** TGFβ2 was significantly downregulated by all treatments involving FGF4. **(C)** All treatments except loading significantly downregulated tenomodulin (Tnmd). **(D)** Collagen type I (Col I) was significantly downregulated by FGF4 and FGF4 + loading, while all treatments involving TGFβ2 caused Col I to trend up (*P* ≥ 0.06). **(E)** Elastin (Eln) was significantly downregulated by all treatments. ↑ or ↓ indicates statistically significant up- or downregulation, respectively; *P < 0.05, **P < 0.01, ***P < 0.001.

MSC number did not change with treatment or time (*P* > 0.05; Figure 1A). MSCs appeared fibroblastic with all treatments (not shown). On D3, Scx was downregulated by FGF4 and FGF4 + loading, but upregulated by TGFβ2 and TGFβ2 + loading (*P* < 0.05; Figure 2A). FGF4 combinations downregulated TGFβ2 (*P* < 0.05; Figure 2B). All combinations, except loading alone, downregulated Tnmd (*P* < 0.05; Figure 2C). FGF4 and FGF4 + loading downregulated Col I (*P* < 0.05). TGFβ2 combinations caused Col I to trend up (*P* > 0.05; Figure 2D), with TGFβ2 + loading approaching significance (*P* = 0.06). All treatments downregulated Eln at D3 compared to control (*P* < 0.05; Figure 2E).

### Effects of treatments on tendon progenitor cells

**Figure 3:**
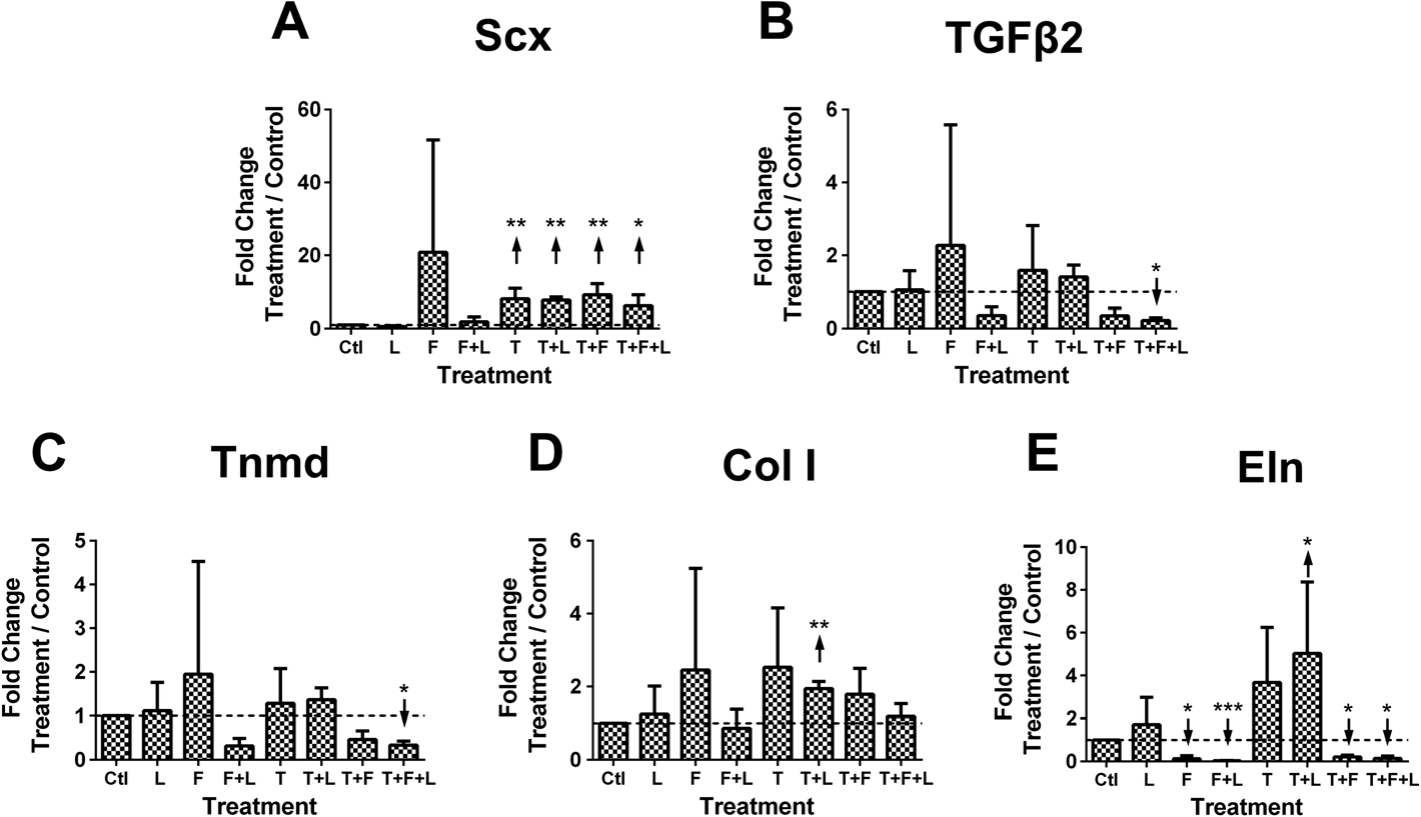
Tendon progenitor cell (TPC) tendon marker gene expression as a function of growth factor treatments and loading. TPC gene expression on day (D)3 of treatment with combinations of mechanical loading (L), transforming growth factor (TGF)β2 (T), and fibroblast growth factor-(FGF)4 (F). Dashed horizontal line = 1 indicates control condition. **(A)** Scleraxis (Scx) was significantly upregulated by all treatments involving TGFβ2. **(B)** TGFβ2 and **(C)** tenomodulin (Tnmd) were significantly downregulated by TGFβ2 + FGF4 + loading. **(D)** Collagen type I (Col I) was significantly upregulated by TGFβ2 + loading. **(E)** Elastin (Eln) was significantly downregulated by all treatments that involve FGF4, but was significantly upregulated by TGFβ2 + loading. ↑ or ↓ indicates statistically significant up- or downregulation, respectively; *P < 0.05, **P < 0.01, ***P < 0.001.

TPC number did not change with treatment or time (*P* > 0.05; Figure 1B), though was higher for TGFβ2 + FGF4 + loading compared to loading alone on D3 (*P* < 0.05; Figure 1B). TPCs appeared fibroblastic with all treatments (not shown). On D3, Scx was upregulated by TGFβ2 combinations (*P* < 0.05), but was not affected by loading, FGF4, or FGF4 + loading (Figure 3A). TGFβ2 and Tnmd were downregulated by TGFβ2 + FGF4 + loading (*P* < 0.05; Figure 3B,C), and exhibited similar expression patterns with all treatments (Figure 3B,C). Col I was upregulated by TGFβ2 + loading (*P* < 0.01), but not affected by other treatments (Figure 3D). Eln was downregulated by FGF4 combinations, but upregulated by TGFβ2 + loading (*P* < 0.05; Figure 3E).

### Comparison of mesenchymal stem cell and tendon progenitor cell gene expression

**Figure 4:**
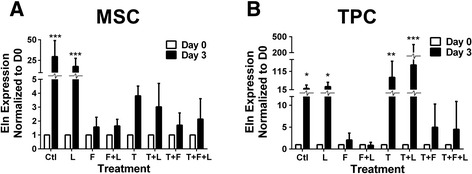
Elastin (Eln) gene expression as a function of growth factor treatments and loading. Eln gene expression in mesenchymal stem cells (MSCs) and tendon progenitor cells (TPCs) on day (D)3 of treatment with combinations of mechanical loading (L), transforming growth factor (TGF)β2 (T), and fibroblast growth factor-4 (F), and normalized to D0. **(A)** MSCs significantly increased Eln with time in control culture and with loading. **(B)** TPCs significantly increased Eln with time in control culture and treatment with loading, TGFβ2, and TGFβ2 + loading. ↑ or ↓ indicates statistically significant up- or downregulation, respectively; *P < 0.05, **P < 0.01, ***P < 0.001.

Under control conditions and when loaded, Eln expression in MSCs increased >20-fold from D0 to D3 (*P* < 0.001; Figure 4A). Other treatment combinations had inhibitory effects on this upregulation of Eln expression (Figure 4A). In TPCs, Eln expression increased >15-fold from D0 to D3 in control culture and with loading (*P* < 0.05; Figure 4B). This increase was abrogated by FGF4 combinations. In contrast to MSCs, TGFβ2 and TGFβ2 + loading enhanced Eln expression in TPCs from D0 to D3 (*P* < 0.01; Figure 4B). Expression of other genes did not vary with time (not shown).

**Figure 5:**
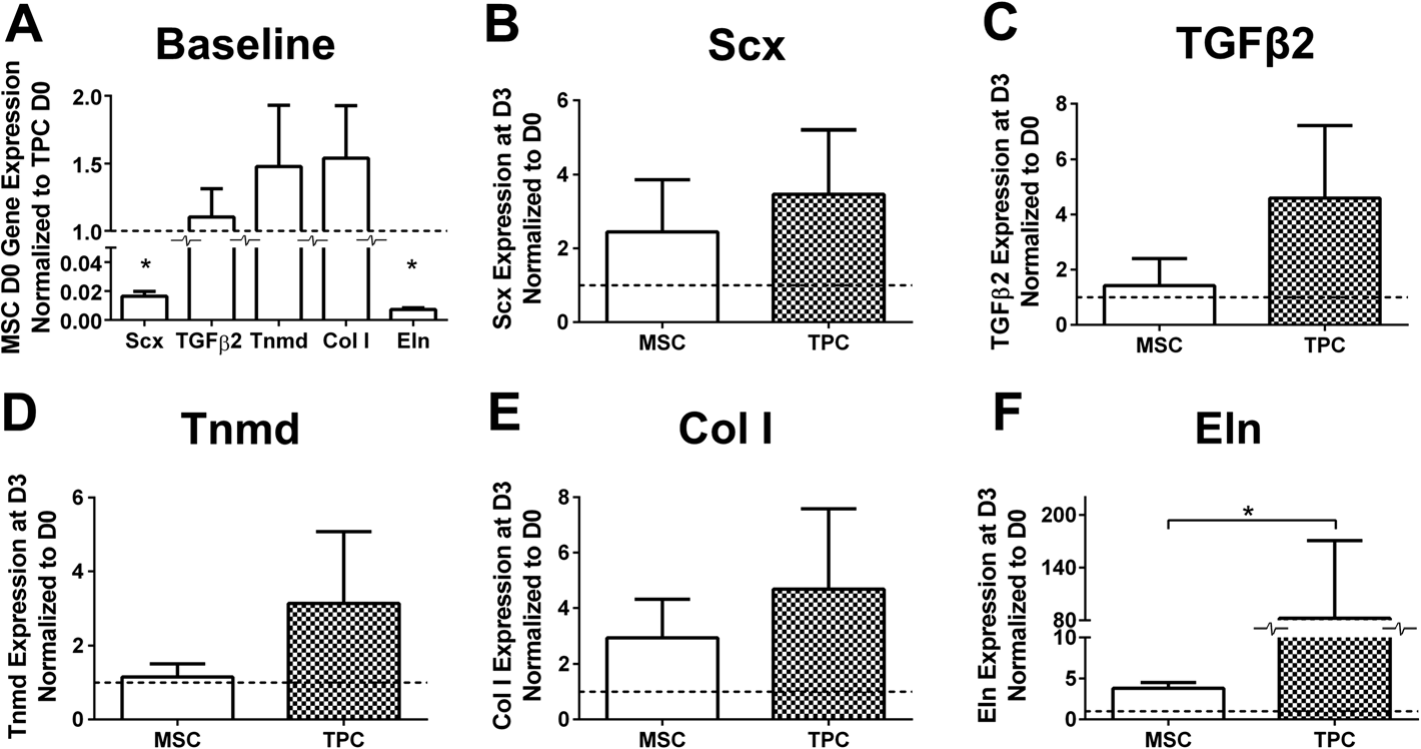
Comparison of mesenchymal stem cell (MSC) and tendon progenitor cell (TPC) tendon marker gene expression at baseline and with transforming growth factor (TGF)β2 treatment. Comparison of MSC and TPC gene expression at day (D)0 and D3 of TGFβ2 treatment. **(A)** Comparison of tenogenic gene expression by MSCs versus TPCs at D0; scleraxis (Scx) and elastin (Eln) were significantly lower in MSCs compared to TPCs. At D3 (normalized to D0) of TGFβ2 treatment, **(B)** Scx, **(C)** TGFβ2, **(D)** tenomodulin (Tnmd), and **(E)** collagen type I (Col I) were not significantly different between MSCs and TPCs, while **(F)** Eln was significantly higher in TPCs than MSCs. *P < 0.05.

To investigate baseline differences between MSCs and TPCs, tendon marker expression levels were compared at D0. Scx and Eln expression in MSCs were lower (61-fold and 138-fold, respectively) than in TPCs (*P* < 0.05; Figure 5A). However, TGFβ2, Tnmd, and Col I levels were similar between TPCs and MSCs (*P* = 0.82, *P* = 0.46 and *P* = 0.36, respectively; Figure 5A). To assess tenogenic potential of MSCs, MSC response to TGFβ2 was compared to that of TPCs at D3 (Figure 5B-F). TGFβ2 was chosen as a tenogenic factor for upregulating Scx in TPCs. On D3 of TGFβ2 treatment, Scx, Col I, TGFβ2, Tnmd and Eln expression trended up in both MSCs and TPCs, compared to D0. MSCs and TPCs were not significantly different in Scx (*P* = 0.54), Col I (*P* = 0.39), TGFβ2 (*P* = 0.13) and Tnmd (*P* = 0.17) levels (Figure 5B-E), but the TGFβ2-induced increases in Eln were 21-fold greater in TPCs than in MSCs (*P* < 0.05; Figure 5F).

## Discussion

To date, a standard protocol to tenogenically differentiate MSCs has not been established. A major challenge is a need for potent tenogenic factors. Another significant obstacle is a lack of metrics to assess tenogenic state. We characterized the effects of embryonic tendon cues on adult MSCs in comparison with embryonic TPCs. Utilizing murine cells enabled investigation of embryonic TPCs, which would not be possible with human cells. Additionally, the murine system develops more rapidly than human. Previous work demonstrated chondrogenic growth factors induced murine embryonic limb bud cells to form Sox9-expressing aggregates in 24 hours, and sulfated proteoglycan-rich cartilaginous nodules by 3 days [41]. Here, embryonic tendon development factors influenced both MSCs and E14 TPCs within 3 days. At E14, TPCs have just condensed into overt tendon tissue forms *in vivo* [42], and embryonic muscle-induced movements that could impose mechanical stimulation on developing tendon are first observed [43]. Our results suggest MSCs have tenogenic potential, based on similar responses as E14 TPCs when subjected to tenogenic treatments.

MSCs and E14 TPCs were examined at D0 to compare baseline gene expression profiles (Figure 5). At D0, cells have been expanded *in vitro* but not subjected to treatments, representing a starting point in many tissue engineering strategies. Tnmd and Col I are late markers of tendon development, so it was expected that MSCs and TPCs in early differentiation stages expressed both genes similarly. Lower baseline Scx levels in MSCs compared to E14 TPCs suggest MSCs are inherently less committed to the tenogenic lineage than TPCs, while similar TGFβ2 and Tnmd levels may reflect that E14 TPCs and MSCs are both at an immature stage of differentiation. TGFβ2 may be a tendon marker as well as a tenogenic cue. TGFβ2 is present in embryonic chick tendons [36], expressed in murine E12.5 to E15.5 limb tendons [37,44], and can upregulate Scx expression in embryonic murine limbs *in vivo* [37]. Additionally, TGFβ2^−/−^ mice possess tendon defects [37].

Cyclic loading alone did not upregulate most tendon markers examined, but increased Scx and Col I expression when combined with TGFβ2 (Figures 2 and 3). Our study was performed with 1% FBS. In contrast, studies in which dynamic loading enhanced Scx and Col I expression in MSCs used significantly higher serum levels [6,8,10,26]. Loading likely interacted with soluble factors from the serum to influence cell behavior in those studies. It is also possible that different loading parameters could yield different results, though others found that changing duty cycle did not affect MSC gene expression of Col I and other extracellular matrix components [4]. Furthermore, we characterized gene expression, which may not reflect changes at the protein level. We previously found cyclic loading enhanced collagen production by human MSCs in three-dimensional scaffolds without altering collagen mRNA levels [7]. Future studies that incorporate three-dimensional culture systems and assess protein level changes will be important.

Both cell types did not change in cell number with treatments (Figure 1) suggesting the treatments were possibly influencing cell functions other than proliferation, such as differentiation. TGFβ2 and TGFβ2 + loading induced higher Scx expression in both MSCs and TPCs when compared to control conditions on D3, though to different levels (Figures 2A and 3A). Conditions that upregulated Scx did not consistently upregulate other tendon genes, including Col I and Tnmd, a reasonable finding as collagen and Tnmd appear in significant amounts later in embryonic development [45-47]. Over time, from D0 to D3, TGFβ2 treatment induced similar trends in Scx, Col I, TGFβ2, and Tnmd expression by MSCs and TPCs (Figure 5B-E). Similar responsiveness of MSCs as TPCs to TGFβ2 treatments compared to control conditions (Figures 2A and 3A) and over time (Figure 5B-E) supports our hypothesis and suggests MSCs have tenogenic potential.

In contrast to TGFβ2, FGF4 combinations downregulated Scx in MSCs (Figure 2A) and had no effect on TPCs (Figure 3A). FGF signaling seems necessary for embryonic tendon development [33,38]. Thus, it was surprising that FGF4 reduced tenogenic marker levels in MSCs. Perhaps differences in composition of transcriptional regulators in MSCs versus TPCs resulted in different signaling responses to the same cues. Potential effects of a heterogeneous progenitor cell population in MSCs should also be considered. Elucidation of these differences could lead to informed tenogenesis strategies using MSCs.

Differential baseline Eln expression levels and responses to TGFβ2 and TGFβ2 + loading by TPCs compared to MSCs are intriguing (Figures 4 and 5). Eln is important for adult tendon function, but little is known about its involvement in tendon development. In our earlier studies, Eln fibers were not detected in embryonic limb tendon [46], though tropoelastin was found in embryonic ligamentum flavum [48]. It would be interesting to investigate Eln influences and elaboration in embryonic tendon development.

## Conclusion

In summary, we showed MSCs have tenogenic potential, based on similar gene expression and proliferation responses as TPCs when subjected to tenogenic treatments. Distinctly parallel trends in gene responses seen with MSCs compared to TPCs suggest the cells share certain molecular mechanisms of responses, which deserve further investigation. Future studies over longer time points could identify TGFβ2 combinations that effectively tenogenically differentiate stem cells, and may elucidate a chronological order of tendon marker expression during tenogenesis. Our findings suggest continued investigation of MSC function in relation to embryonic TPCs could contribute to advancements in tendon tissue regeneration strategies.

## Note

This article is part of an ‘Emerging Investigators’ collection showcasing the work of early career investigators who have demonstrated growing leadership in the field of stem cells and regenerative medicine. Other articles in the series can be found online at http://stemcellres.com/series/emerginginvestigators

## Box 1. About Catherine K. Kuo

**CKK** is an Assistant Professor of Biomedical Engineering at Tufts University, and a faculty member of the Cell, Molecular and Developmental Biology Program in the Sackler School of Graduate Biomedical Sciences at the Tufts University School of Medicine. She earned a BSE in Materials Science and Engineering and a PhD in Biomaterials and Macromolecular Science and Engineering from the University of Michigan, and pursued postdoctoral training in the Cartilage Biology and Orthopaedics Branch of the NIAMS at the National Institutes of Health. Her research focuses on developing adult stem cell differentiation strategies informed by embryogenesis. Specifically, she is identifying mechanical and chemical properties of embryonic tissue microenvironments that can be presented via biomaterials and bioreactor cultures to direct stem cell behavior. With this approach, she is designing replacement tissues for regenerative medicine, and also engineering tissue models as platforms to investigate mechanisms of embryonic tissue formation and wound healing.

## Abbreviations

BM: basal medium
Col I: collagen type I
D: day
E: embryonic day
Eln: elastin
FBS: fetal bovine serum
FGF: fibroblast growth factor
GFP: green fluorescent protein
GM: growth medium
MSC: mesenchymal stem cell
PBS: phosphate-buffered saline
qPCR: quantitative polymerase chain reaction
Scx: scleraxis
TGF: transforming growth factor
Tnmd: tenomodulin
TPC: tendon progenitor cell
